# FEASIBILITY AND ACCEPTABILITY OF A DEPRESSION AND PAIN INTERVENTION WITH OLDER AFRICAN AMERICAN WOMEN

**DOI:** 10.1093/geroni/igad104.1023

**Published:** 2023-12-21

**Authors:** Janiece Taylor, Catherine Clair, Laura Gitlin, Karen Bandeen-Roche, Melissa deCardi Hladek, Tiffany Riser, Roland Thorpe, Sarah L Szanton

**Affiliations:** Johns Hopkins University, Baltimore, Maryland, United States; Johns Hopkins Bloomberg School of Public Health, Baltimore, Maryland, United States; Drexel University, Philadelphia, Pennsylvania, United States; Johns Hopkins University, Baltimore, Maryland, United States; Johns Hopkins School of Nursing, Baltimore, Maryland, United States; John Hopkins School of Nursing, Baltimore, Maryland, United States; Johns Hopkins University, Baltimore, Maryland, United States; Johns Hopkins University, Baltimore, Maryland, United States

## Abstract

Older African American women experience social determinants of health that put them at risk for experiencing comorbid pain and depressive symptoms. The purpose of our study was to tailor a preexisting evidence-based depression intervention to include pain and test it in older African American women. We conducted a randomized waitlist control study with 21 frail or pre-frail, African American women, 50 years of age and older with pain and depressive symptoms. The average age of the participants was 64.8 (SD: 10.5), average pain intensity was 7.0 (SD: 1.9) out of 10, and average PHQ-9 depressive symptoms score was11.9 (SD: 4.0). Effect sizes at 12 weeks post intervention were -1.05 for depressive symptoms indicating a substantial decrease in depressive symptoms. We did not see a significant change in pain intensity but identified changes in pain behavior scores. The women described the intervention as beneficial and provided suggestions for future iterations.

